# Supplementary material for: Polyphasic Approach Utilized for the Identification of Two New Toxigenic Members of Penicillium Section Exilicaulis, P. krskae and P. silybi spp. nov
Source: J Fungi (Basel). 2021 Jul 13;7(7):557. doi: 10.3390/jof7070557 (PMC8307998; doi:10.3390/jof7070557)
Supplement: Supplementary file 1 [file jof-07-00557-s001.zip › jof-1294679-supplementary.pdf]

Journal of *Fungi*

MDPI

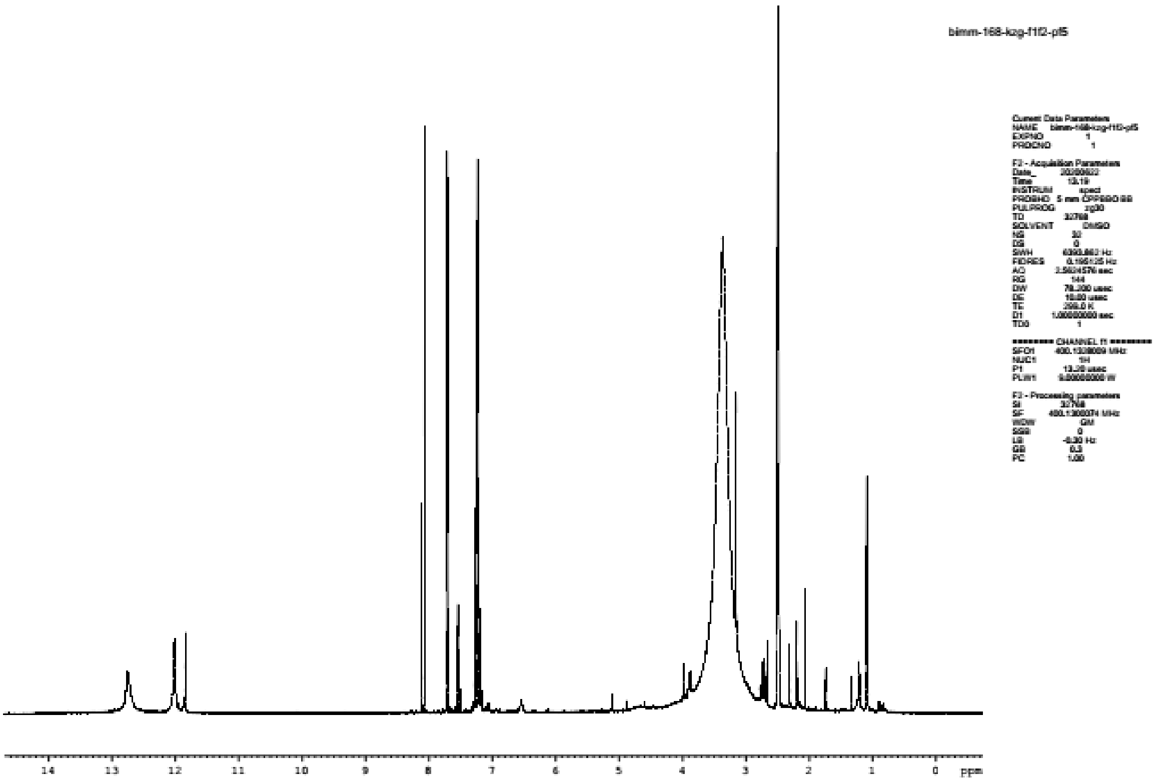

Figure S1. ^{1}H NMR spectrum of the mixture of **1**, **2**, and **3.**

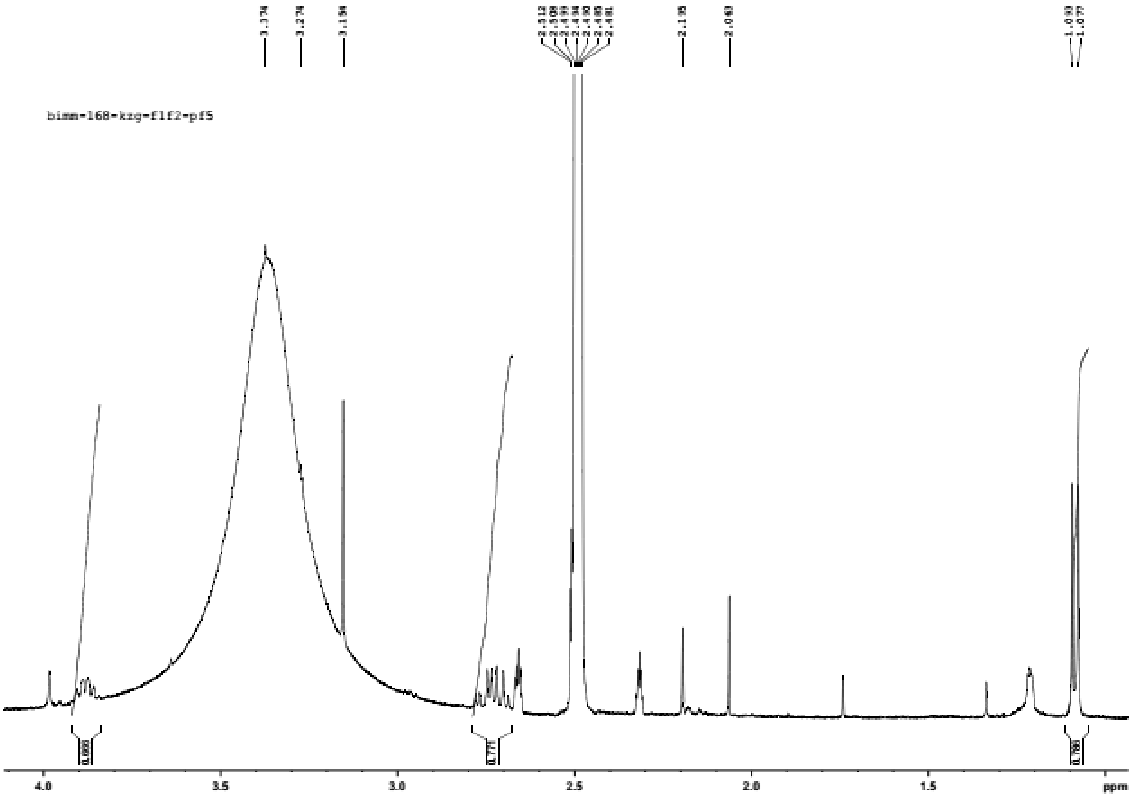

Figure S2. ^{1}H NMR spectrum of the mixture of **1**, **2**, and **3**, expansion.

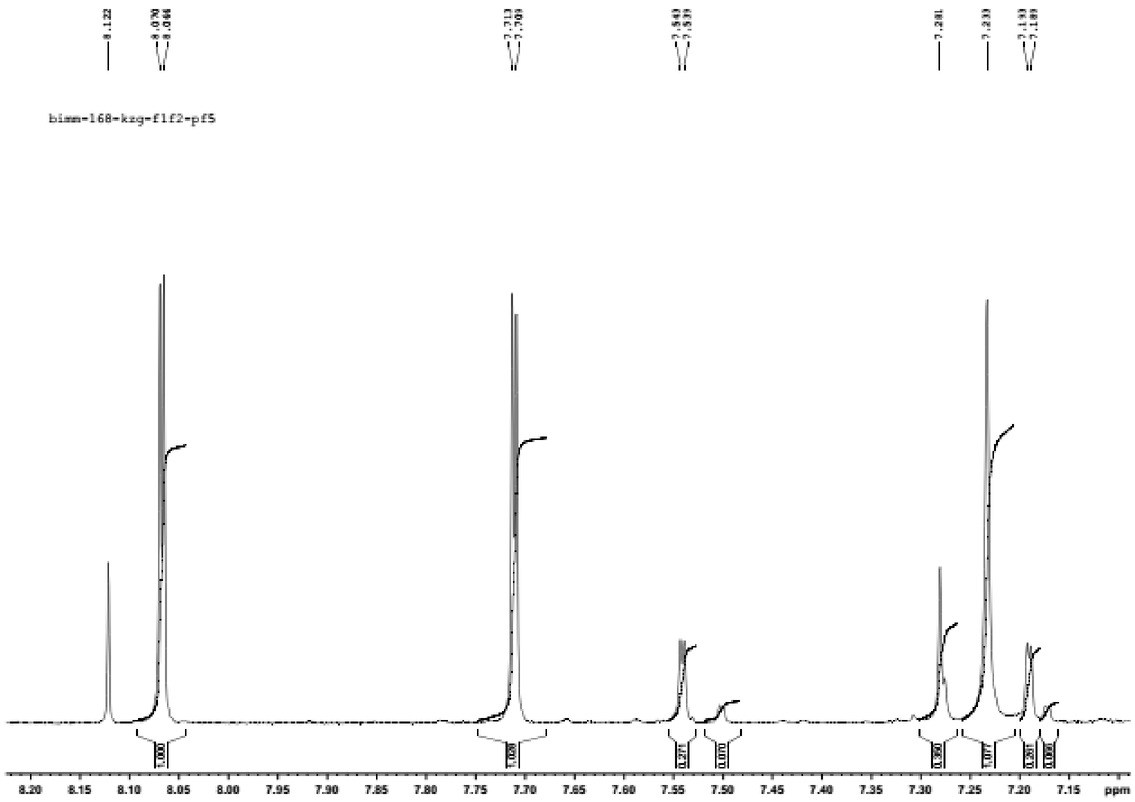

Figure S3. ^{1}H NMR spectrum of the mixture of **1**, **2**, and **3**, expansion.

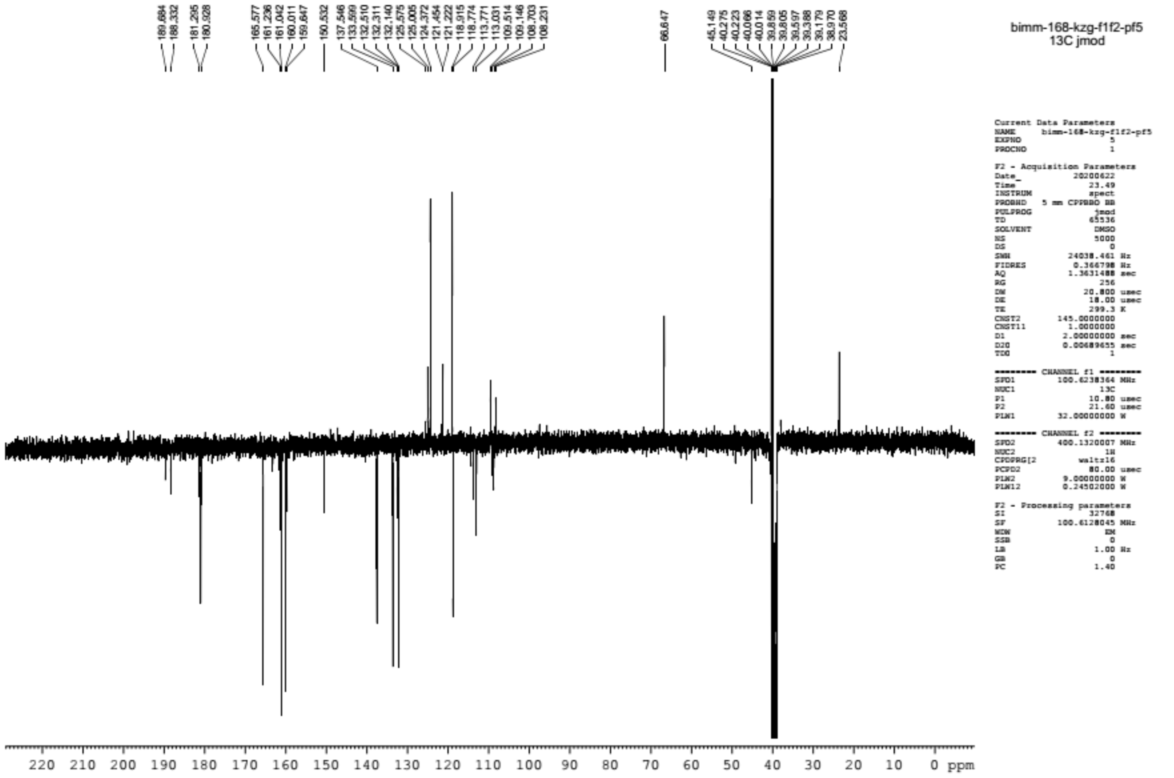

Figure S4. ^{13}C NMR spectrum of the mixture of **1**, **2**, and **3.**

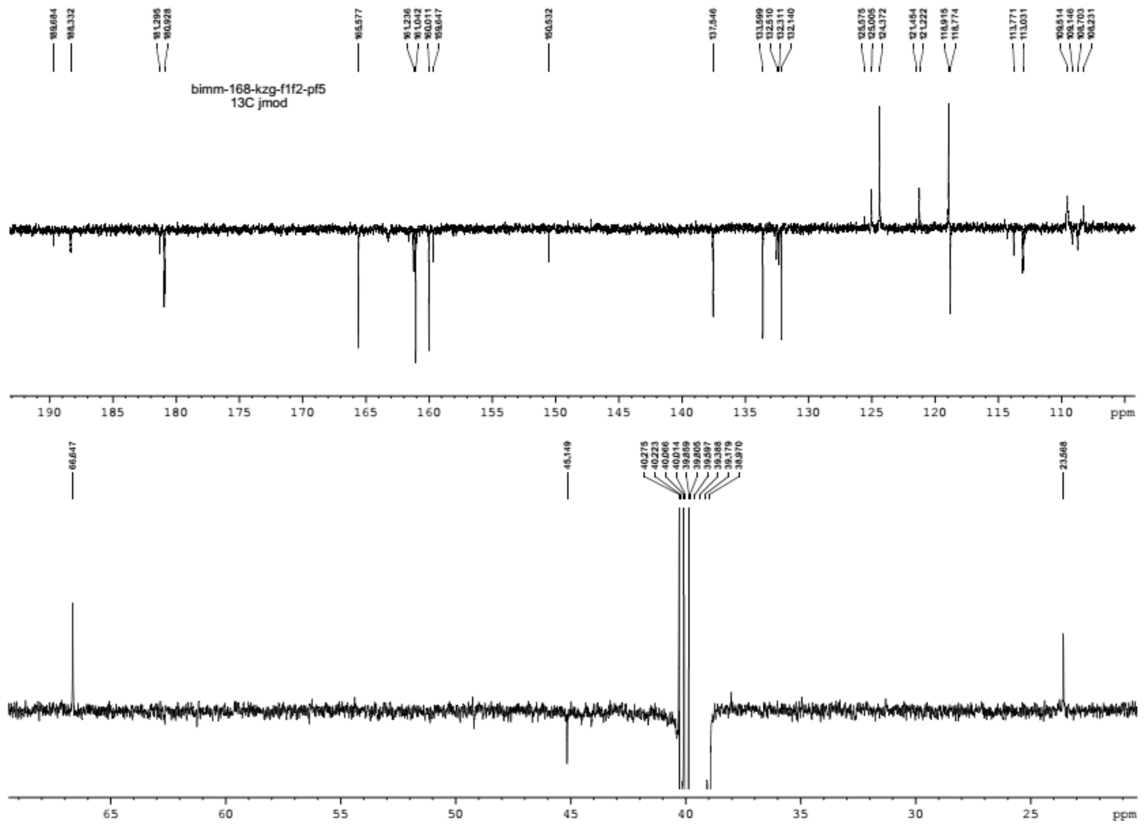

Figure S5. ^{13}C NMR spectrum of the mixture of **1**, **2**, and **3**, expansion.

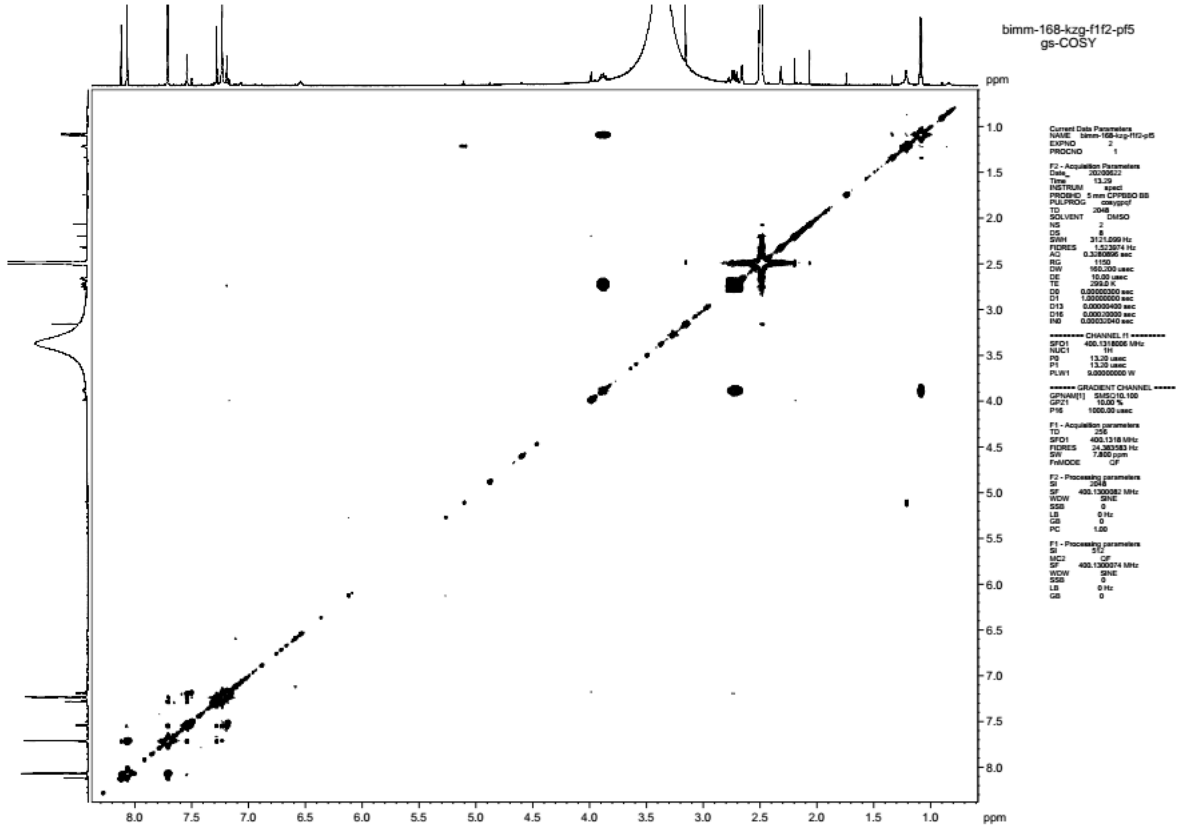

Figure S6. COSY NMR spectrum of the mixture of **1**, **2**, and **3.**

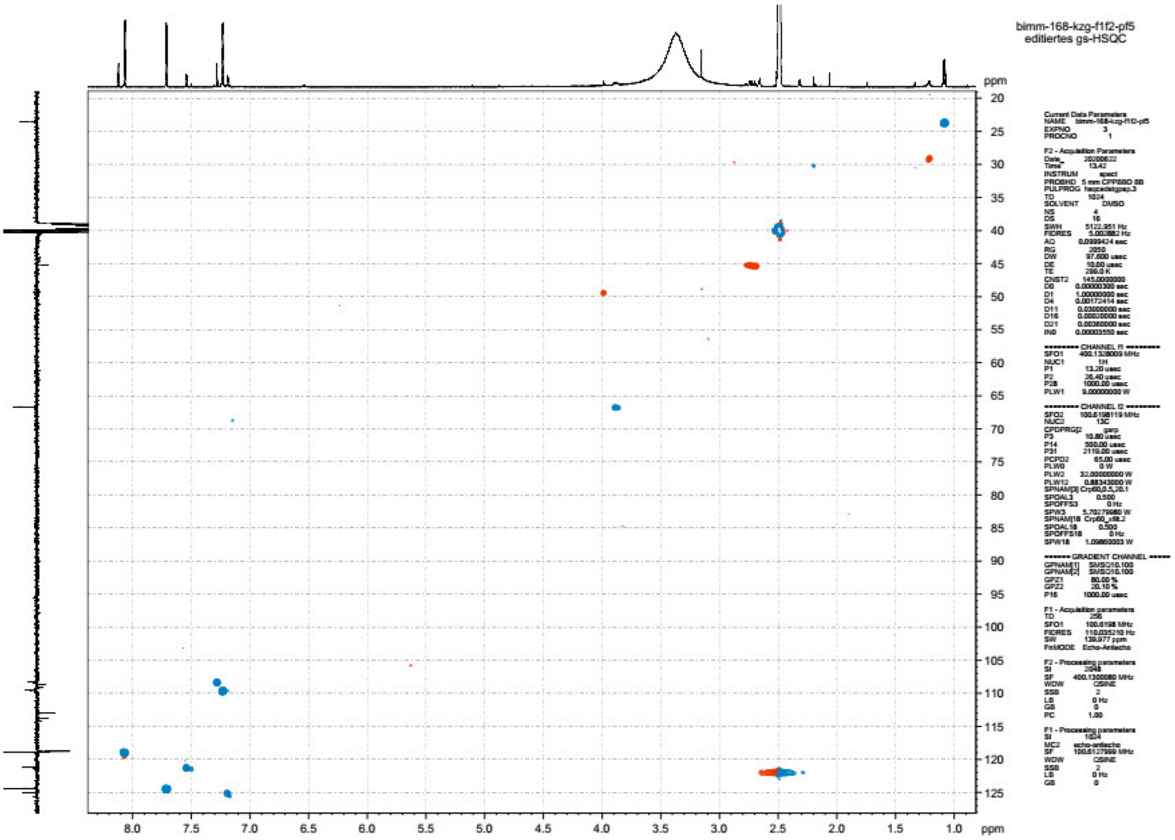

Figure S7. Edited HSQC NMR spectrum of the mixture of **1**, **2**, and **3.**

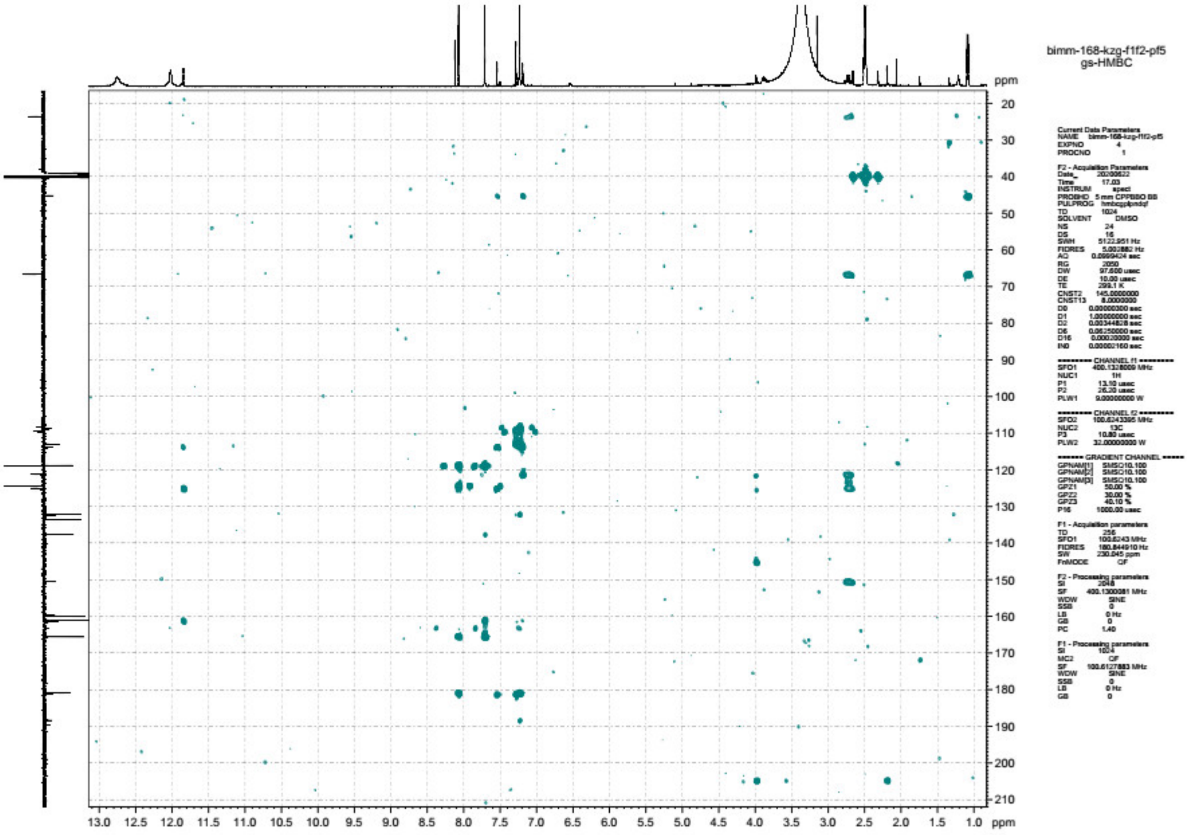

Figure S8. HMBC NMR spectrum of the mixture of **1**, **2**, and **3.**

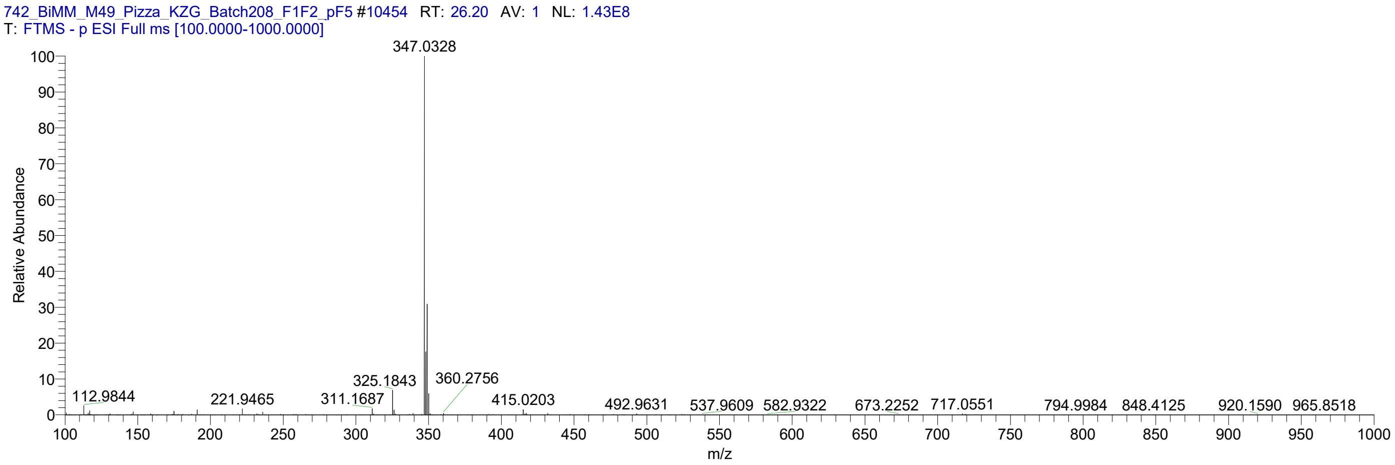

Figure S9. LC-ESI-MS spectrum negative mode (26.2 min) of compound **1.**

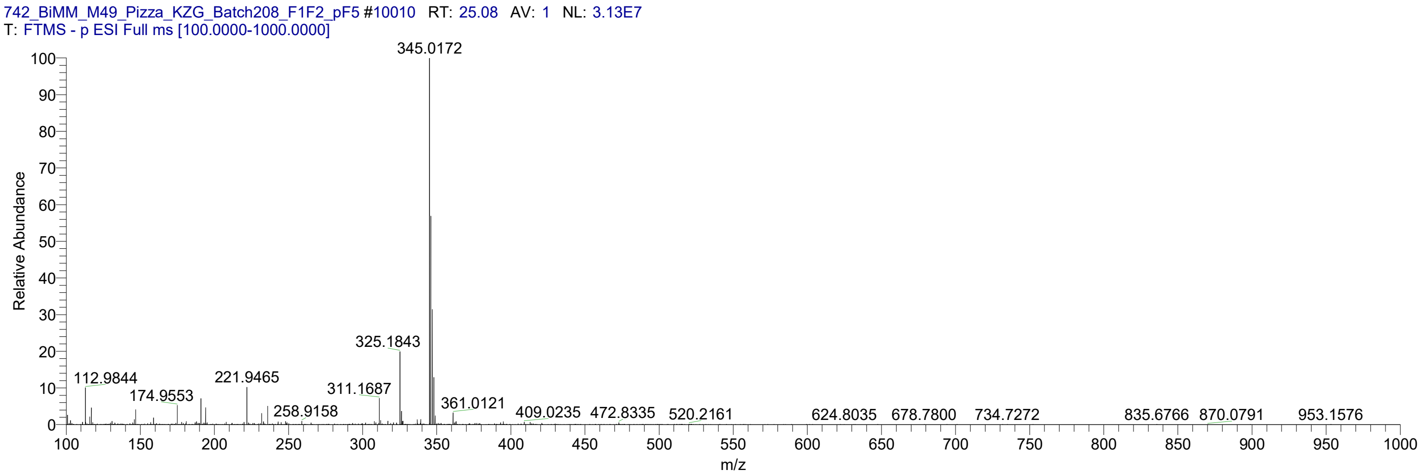

Figure S10. LC-ESI-MS spectrum negative mode (25.1 min) of compound **2.**

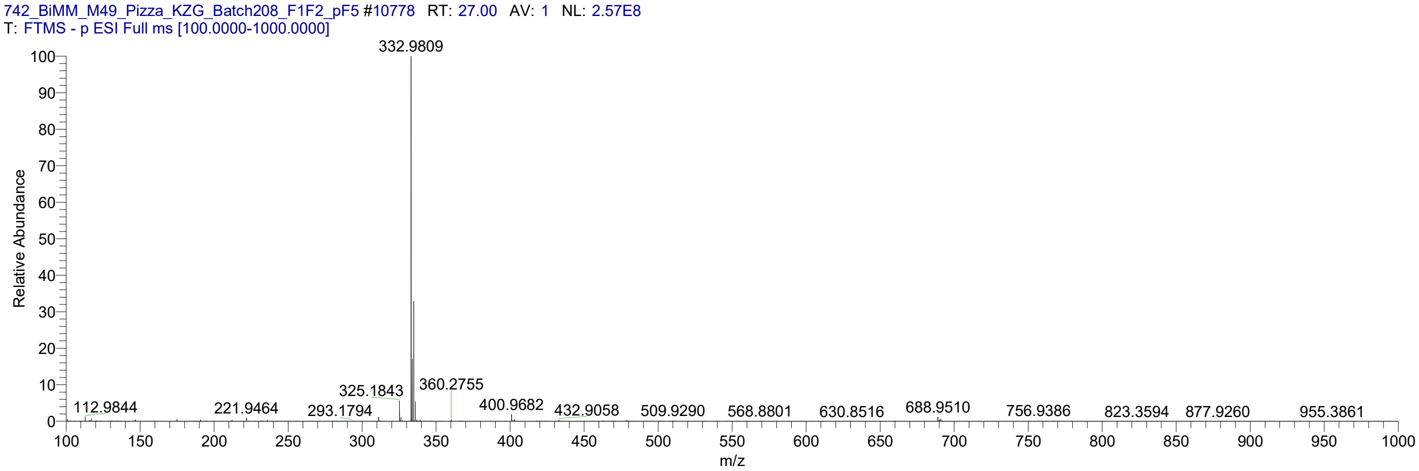

Figure S11. LC-ESI-MS spectrum negative mode (27.0 min) of compound **3.**

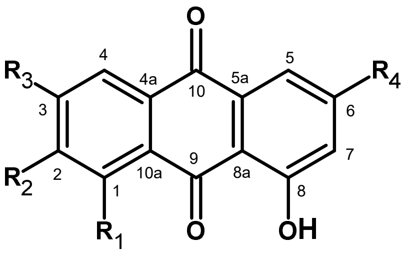

	R_1	R_2	R_3	R_4
**2-chloro-isorhodoptilometrin (1)**	OH	Cl	OH	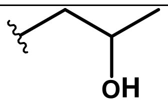
**2-chloro-desmethyl dermoquinone (2)**	OH	Cl	OH	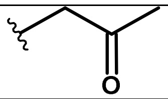
**2-chloroemodic acid (3)**	OH	Cl	OH	**COOH**
**7-chlorocitrorosein**	OCH_3	Cl	OH	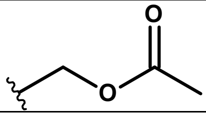
**emodin**	OH	H	OH	**CH_3**
**citreorosein (ω-hydroxyemodin)**	OH	H	OH	**CH_2OH**
**emodic acid**	OH	H	OH	**COOH**
**(+)-2′*S*-isorhodoptilometrin**	OH	H	OH	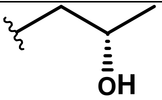
**1′-hydroxyisorhodoptilometrin**	OH	H	OH	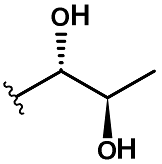
**1′-hydroxy-2′-keto-isorhodoptilometrin**	OH	H	OH	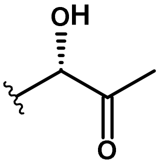
**desmethyl dermoquinone**	OH	H	OH	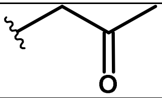
**dermoquinone**	OH	H	OCH_3	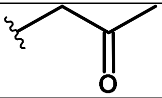
**2-hydroxyemodic acid**	OH	OH	OH	**COOH**

Figure S12. Structures of detected anthraquinone derivatives.

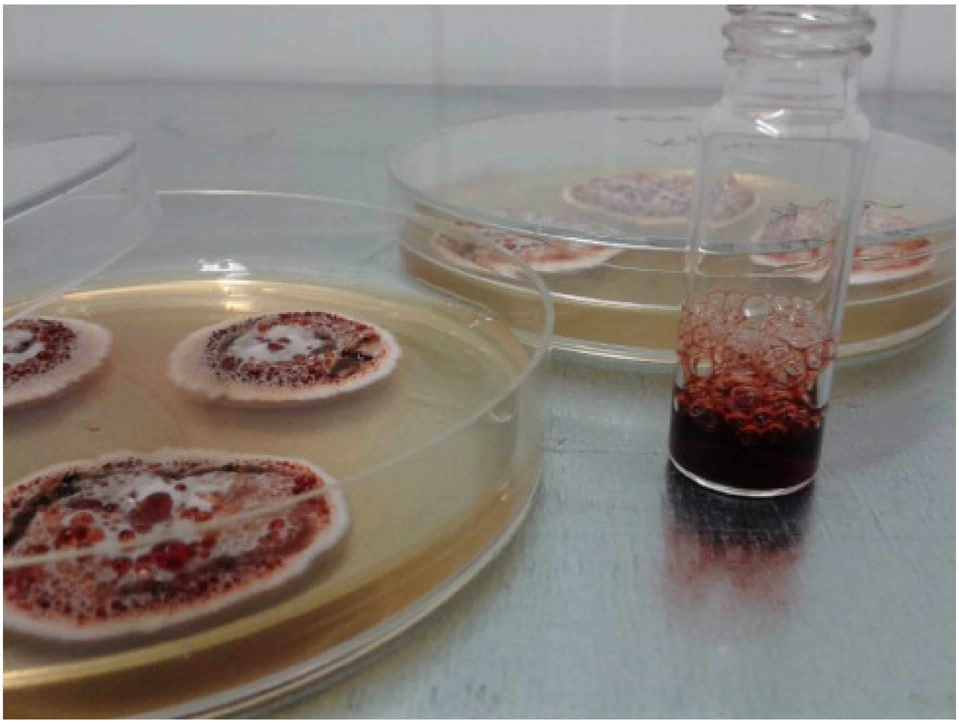

Figure S13. Colonies of *P. krskae* BiMM-F280 growing on MEA (7days, 25°C, dark) and red exudate collected for LC-MS/MS analysis.
